# Plasticity in Limbic Regions at Early Time Points in Experimental Models of Tinnitus

**DOI:** 10.3389/fnsys.2019.00088

**Published:** 2020-01-24

**Authors:** Michelle R. Kapolowicz, Lucien T. Thompson

**Affiliations:** ^1^Center for Hearing Research, University of California, Irvine, Irvine, CA, United States; ^2^Department of Otolaryngology-Head and Neck Surgery, School of Medicine, University of California, Irvine, Irvine, CA, United States; ^3^Department of Neurobiology, School of Behavioral and Brain Sciences, The University of Texas at Dallas, Richardson, TX, United States

**Keywords:** tinnitus, amygdala, hippocampus, cingulate cortex, noise trauma, salicylate

## Abstract

Tinnitus is one of the most prevalent auditory disorders worldwide, manifesting in both chronic and acute forms. The pathology of tinnitus has been mechanistically linked to induction of harmful neural plasticity stemming from traumatic noise exposure, exposure to ototoxic medications, input deprivation from age-related hearing loss, and in response to injuries or disorders damaging the conductive apparatus of the ears, the cochlear hair cells, the ganglionic cells of the VIIIth cranial nerve, or neurons of the classical auditory pathway which link the cochlear nuclei through the inferior colliculi and medial geniculate nuclei to auditory cortices. Research attempting to more specifically characterize the neural plasticity occurring in tinnitus have used a wide range of techniques, experimental paradigms, and sampled at different windows of time to reach different conclusions about why and which specific brain regions are crucial in the induction or ongoing maintenance of tinnitus-related plasticity. Despite differences in experimental methodologies, evidence reveals similar findings that strongly suggest that immediate and prolonged activation of non-classical auditory structures (i.e., amygdala, hippocampus, and cingulate cortex) may contribute to the initiation and development of tinnitus in addition to the ongoing maintenance of this devastating condition. The overarching focus of this review, therefore, is to highlight findings from the field supporting the hypothesis that abnormal early activation of non-classical sensory limbic regions are involved in tinnitus induction, with activation of these regions continuing to occur at different temporal stages. Since initial/early stages of tinnitus are difficult to control and to quantify in human clinical populations, a number of different animal paradigms have been developed and assessed in experimental investigations. Reviews of traumatic noise exposure and ototoxic doses of sodium salicylate, the most prevalently used animal models to induce experimental tinnitus, indicate early limbic system plasticity (within hours, minutes, or days after initial insult), supports subsequent plasticity in other auditory regions, and contributes to the pathophysiology of tinnitus. Understanding this early plasticity presents additional opportunities for intervention to reduce or eliminate tinnitus from the human condition.

## Introduction

Tinnitus is described as a perception of sound(s), such as ringing, buzzing, or hissing, when no external sound is present. Tinnitus is the most widespread auditory disorder, steadily growing in incidence due to a rise in traumatic noise exposure (e.g., from combat, recreation, and work) and to an increase in the aging population (Rauschecker et al., 2010). Despite a growing amount of research effort focused on tinnitus, there still remains no consistent treatment or cure for this condition.

Systems-level approaches using neurophysiological and imaging techniques have shown numerous brain regions exhibit hyperactivity in tinnitus, in both classic lemniscal auditory regions (Arnold et al., 1996; Melcher et al., 2009) as well as in non-classic regions (Lockwood et al., 1998; Schlee et al., 2009) in human patients diagnosed with tinnitus. Despite the wide range of brain regions and networks seemingly altered in tinnitus, there is considerable disagreement in the literature as to where tinnitus initially manifests; i.e., there is no consensus as to the specific mechanisms or loci involved in the generation of tinnitus. While it is widely agreed that tinnitus is often triggered by cochlear damage resulting in maladaptive plasticity in the central auditory system (Mühlnickel et al., 1998), general scientific or clinical consensus regarding the consequences of this maladaptation has not emerged.

Overwhelming evidence shows that sensorineural hearing loss caused by either noise trauma (Dobie, 2008; Kujawa and Liberman, 2009) or exposure to ototoxic drugs, such as salicylate (Stypulkowski, 1990; Bisht and Bist, 2011; Yorgason et al., 2011), may result in a reduction in transmission of neural activity from the cochlea to the central auditory system. Consequently, activity in the central auditory system is enhanced at suprathreshold intensities. This compensatory increase in the central auditory system due to the loss of sensory input led to the dominant “central gain enhancement hypothesis” as a means to explain a potential mechanism for tinnitus induction (for a review, see Auerbach et al., 2014). Notably, Auerbach et al. (2014) also discuss central gain enhancement in non-lemniscal limbic regions, including the amygdala. Since debilitating tinnitus is often accompanied by negative emotions including anxiety, stress, depression, and sleep disturbances (Rizzardo et al., 1998), it is not surprising that evidence has accumulated showing that the amygdala, in addition to classic auditory structures, can be involved in tinnitus (e.g., Crippa et al., 2010). The amygdala has been widely accepted for its role in processing aversive auditory stimuli (e.g., Zald and Pardo, 2002), though alternative findings have been reported regarding auditory fear conditioning (Weinberger, 2011). The amygdala has reciprocal connections to the medial geniculate nucleus (MGN) and auditory cortex (A1; LeDoux and Farb, 1991). For example, it has been shown that when salicylate is applied locally to the amygdala, local field potential (LFP) responses in the A1 are greatly enhanced (Chen et al., 2012), consistent with the notion of central gain enhancement involving a limbic region of the brain.

**Figure 1:**
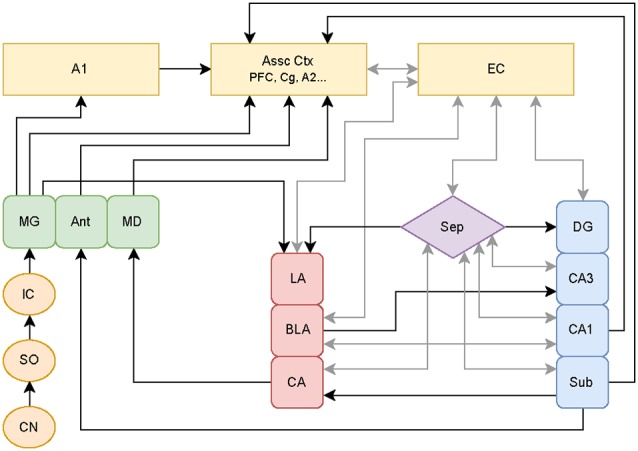
Schematic of classical and non-classical auditory pathways. The medial geniculate nucleus of the thalamus (MG) can follow the classical auditory pathway to the primary auditory cortex (A1), or it can branch off to directly activate limbic pathways. These pathways may be stimulated simultaneously with parallel processing occurring between the amygdala-hippocampal circuits. Additional abbreviations: cochlear nucleus (CN), superior olive (SO), inferior colliculus (IC), anterior (Ant), medial dorsal (MD) thalamic nuclei, association cortices (Assoc Ctx), prefrontal cortex (PFC), cingulate (Cg), secondary auditory cortex (A2), entorhinal cortex (EC), septum (Sep), lateral (LA), basolateral (BLA), and central (CA) amygdaloid nuclei, and hippocampal subregions consisting of dentate gyrus (DG), cornu ammonis 3 (CA3), cornu ammonis 1 (CA1), subiculum (Sub). Circuitry compiled from established auditory-limbic connections as reported in Møller et al. (1992); Møller and Rollins (2002) and Kandratavicius et al. (2012) using draw.io online software (www.draw.io).

Other hypotheses involving the aberrant filtering of auditory information by limbic regions have been put forth to explain the origin of tinnitus. Jastreboff and Jastreboff (2000) proposed that limbic hyperactivity observed in tinnitus patients plays a limited role, with this hyperactivity specifically causing the emotional reactions in tinnitus. However, Rauschecker et al. (2010) proposed that if limbic structures fail to block hyperactive signals generated in classic auditory structures, this “filter failure” leads to chronic forms of tinnitus. Both models assume that cortical regions are responsible for the origin of tinnitus. The latter model also asserts that certain limbic regions are responsible for the ability to cancel the tinnitus percept. Specifically, certain limbic regions may serve an inhibitory gating role for tinnitus perception by functioning as part of a feedback pathway from the amygdala to the auditory system. This inhibition may suppress tinnitus subcortically prior to reaching the A1 and consciousness. Rauschecker et al. (2010) focused on data from various human imaging studies, which give a clear picture of brain states well after the initial stages of tinnitus, but do not reflect potential plastic changes occurring at much earlier time points that can contribute to the initiation as well as the maintenance of tinnitus. Since humans do not present clinically until the disorder is readily apparent, assessment of early plasticity is best characterized in animal models of tinnitus (see Figure 1 for diagram showing established connections linking known auditory structures with limbic structures).

Kraus and Canlon (2012) elaborated on the importance of limbic involvement, citing studies reporting evidence for reciprocal interactions between auditory areas and limbic regions contributing to the generation of tinnitus (e.g., Mühlau et al., 2006). Kraus and Canlon (2012) also discussed the potential role of limbic involvement in the stabilization or cancellation of tinnitus (as proposed by Rauschecker et al., 2010), noting that, since limbic and auditory systems are interconnected, tinnitus can affect emotional as well as cognitive processing, which can, in turn, affect auditory percepts. Kraus and Canlon (2012) focused their review on tinnitus linked to traumatic noise exposure. Although noise trauma is a common cause of tinnitus, it is important to determine whether other common causes of tinnitus, such as ototoxic medications, share similar mechanisms and time courses for development.

Evidence supporting the hypothesis that limbic regions play a strong role in the generation, induction, maintenance, and suppression of tinnitus has accrued, suggesting the limbic system could be considered as a viable target for tinnitus treatment. Three key limbic regions are strongly implicated: the amygdaloid complex, the hippocampus, and the cingulate cortex. This review summarizes plasticity, excitation, and inhibition across early stages of tinnitus pathology resulting from either acute traumatic noise exposure or sodium salicylate treatment (the two most commonly used inductive paradigms in animals) in these key limbic regions. Hyperexcitability and other forms of plasticity within the amygdala, hippocampus, or cingulate cortex often occur immediately after initial exposure to either traumatic noise or to an ototoxic substance, and continues until well after behavioral evidence of tinnitus has been observed. Enhanced excitability within these limbic regions does not always co-occur with markers depicting decreases in inhibition (disinhibition). Remarkably, only a few studies to date have measured potential changes in inhibition in these key limbic regions in models of the development of tinnitus, so it is not currently possible to accurately characterize specific changes in inhibition in the early stages of tinnitus. The available evidence, however, supports the hypothesis that a maladaptive down-regulation of GABAergic neurotransmission occurs throughout the central auditory pathway in tinnitus (Wang et al., 2011; Richardson et al., 2012).

## Animal Models of Tinnitus

Given the growing population of tinnitus sufferers worldwide, it has become essential to develop reliable animal models, both to understand its underlying mechanisms and in hope of developing treatments that can reverse tinnitus’ maladaptive plasticity. Such models permit approaches beyond what is possible in human patients, with experiments capable of addressing individual neurons, reduced or enhanced networks, as well as characterizing the disorder at multiple time points. Animal models allow for use of invasive procedures with high spatial and temporal resolution. As noted, the two most common methods implemented to induce tinnitus in animal models are exposure to traumatic noise and treatment with high doses of sodium salicylate. These methods cause cochlear damage, which triggers a sequence of events leading to the development of tinnitus in both humans and in animal models. Both exposure to traumatic noise and treatment with high doses of sodium salicylate cause tinnitus in human populations, aiding translation from the laboratory to the clinic. For a detailed review of these two models, see von der Behrens (2014).

Traumatic noise exposure is one of the most common risk factors for tinnitus in human populations. Traumatic noise exposure has also been well established to induce tinnitus in animal models (e.g., Brozoski and Bauer, 2005; Turner et al., 2006; Engineer et al., 2011), though the parameters for induction are variable. Typically, a specific tone frequency is played at a high volume for 1–2 h either unilaterally or bilaterally while the animal is under anesthesia. Few studies, however, expose animals while they are freely behaving (which would be more analogous to human exposure conditions), hence the results from the noise trauma exposure are potentially confounded with the effects of anesthesia. For additional information on the role of anesthesia in tinnitus development, see von der Behrens (2014).

Since the probability of developing tinnitus after traumatic noise exposure is relatively inconsistent, researchers have also utilized paradigms consisting of treatment with ototoxic drugs, with sodium salicylate the most commonly used in animal models (Cazals, 2000). High doses of sodium salicylate, the active ingredient in aspirin, can cause temporary hearing loss and also consistently induces reversible tinnitus in both humans and animals (Jastreboff et al., 1988; Day et al., 1989; Jastreboff, 1990; Brien, 1993; Bauer et al., 1999; Chen et al., 2012). Salicylate inhibits cyclooxygenase and stimulates arachidonic acid production, which has been shown to facilitate NMDA receptor-mediated responses to glutamate released spontaneously by inner hair cells (IHC; Ruel et al., 2008). Treatment with salicylate is a useful paradigm to infer whether the neuronal enhancement seen after noise-induced hearing loss is also consistently seen in animals that are experiencing tinnitus, and its effects are rapid (Auerbach et al., 2014). For a thorough review of the salicylate model of tinnitus, see Stolzberg et al. (2012).

Animal models of tinnitus should ideally model human pathogenesis. It is widely suspected that traumatic noise exposure is the most common causative event for human tinnitus populations, which supports using an acoustic trauma paradigm to induce tinnitus in animals in preference over using an ototoxic substance. However, tinnitus induction *via* high doses of salicylate in animals yields a rapid (and higher percentage) onset of tinnitus. Given the widespread use of both methods for tinnitus induction in animal models, this review will cover the neural correlates of both models within the amygdaloid complex, the hippocampus, and the cingulate cortex. Evidence will be summarized supporting the notion that outcomes from both methods of induction are consistently similar at early time points post-treatment. This review will also cover specific cases of animal models of noise trauma exposure which are not directly linked to tinnitus. Given that noise trauma is the most common cause of tinnitus and hearing loss, such animal models are important to be considered given that results from these noise trauma paradigms share similarities with reports from studies where behavioral evidence of tinnitus was also provided. Interestingly, while relatively homogenous outcomes are observed in these limbic structures (discussed below), traumatic noise exposure and salicylate affect classic auditory structures, including the cochlea and the central auditory system, in a much more heterogeneous manner. For more information on the divergence of observed mechanisms and patterns of maladaptive plasticity resulting from these two inductive paradigms in classical auditory pathway regions, see Eggermont (2016).

## Key Limbic Regions Involved in the Pathophysiology of Tinnitus

### Amygdaloid Complex

The amygdaloid complex (amygdala) is noted for processing emotionally salient information (Phelps and LeDoux, 2005). The amygdala is strongly involved in a range of behavioral functions and psychiatric disorders, and it has been implicated in tinnitus (e.g., De Ridder et al., 2006). The amygdala is divided into the lateral (LA), basal (BA), central (CeA) and medial (MeA) nuclei (LeDoux, 2007). The LA receives input from multiple sensory systems; the MeA receives information from the olfactory bulb; the CeA receives input from the viscerosensory cortex and sensory brainstem; the BA receives input from polymodal association cortex areas and from regions processing memory and cognition, i.e., it is linked functionally with the hippocampus (Kraus and Canlon, 2012). The LA is important in sound processing because it receives direct neuronal inputs from the MGN and from secondary auditory association areas (Sah et al., 2003; LeDoux, 2007). Additionally, auditory information reaching the LA through the MGN can signal this region to activate the hippocampus *via* output projections, which influence the sensitivity of neurons in the A1 (Chavez et al., 2009). Amygdalar responses to sound depend on how important the sound is in the individual’s sensory environment (Klinge et al., 2010), and it is sensitive to stimuli with emotional valence (Anders et al., 2008). Activation of BA and LA neurons is critically involved in the maintenance of emotional salience (Sengupta et al., 2018) and in the processing of emotionally salient stimuli by hippocampal neurons (McIntyre et al., 2005; Farmer and Thompson, 2012; Lovitz and Thompson, 2015).

### Hippocampus

The hippocampus is widely known for its involvement in learning and the formation of new memories. It is active in explicit or declarative memory (Dickerson and Eichenbaum, 2010), including episodic and semantic memory. The hippocampus is also involved with spatial memory (Thompson and Best, 1989, 1990; O’Keefe et al., 1998; Goble et al., 2009). Hippocampal synaptic integrity has also been shown to be impaired by hearing loss (Yu et al., 2011), and hippocampal neurogenesis is decreased by acoustic trauma (Liu et al., 2016). The hippocampus consists of several subregions: dentate gyrus (DG), cornu ammonis (CA) CA1, CA2, CA3, CA4 and adjacent subicular subregions. Major excitatory afferents enter the hippocampus from the entorhinal cortex (EC) *via* the perforant path, projecting to granule cells in the DG, which then project *via* the mossy fibers to CA3, with CA3 neurons projecting to CA1 *via* Schaffer collaterals, with major cortical efferents projecting from CA1 neurons; CA1 also projects to the subiculum, which has outputs to subcortical regions (Amaral et al., 2007). Once information leaves the hippocampus (*via* either CA1 or subicular projections), it can be passed directly to the lateral, basal and medial nuclei of the amygdala as well as to its intercalated cells (Kishi et al., 2006; Cenquizca and Swanson, 2007). The amygdala, *via* the lateral or basal nuclei, has direct projections to hippocampus as well *via* CA3, CA1 or the subiculum as well as indirect projections *via* the EC (Kraus and Canlon, 2012). Like the amygdala, the hippocampus also responds to sound by either direct or indirect input from various auditory association cortices (Mohedano-Moriano et al., 2007; Munoz-Lopez et al., 2010). There are also direct connections from CA1 to the auditory association cortex as well as to the primary A1 (Cenquizca and Swanson, 2007) which are involved in the formation of long-term auditory memories (Squire et al., 2001). The hippocampus is also indirectly connected to auditory regions *via* the front medial cortex, the insula and the amygdala (Kraus and Canlon, 2012).

### Cingulate Cortex

The cingulate cortex is involved with emotional responsivity (Hadland et al., 2003; Vogt, 2005). It is also involved in learning (Aly-Mahmoud et al., 2017) and memory (Kozlovskiy et al., 2012). The cingulate has a major role in behavioral drive and regulation of affective behavior, e.g., it is involved in emotional processing and inhibitory control (Shackman et al., 2011; Holloway-Erickson et al., 2012; Xie et al., 2013). The cingulate gyrus is also critically involved in attentional processing and in sleep staging, with greatest activity observed in response to emotionally arousing stimuli in the waking state and during REM sleep periods crucial for memory consolidation (Oniani et al., 1989; Wang and Ikemoto, 2016). Wang and Ikemoto (2016), for example, found an increase in anterior cingulate cortex (ACC) neuronal firing which is influenced by hippocampal ripple activity during sleep and speculated that this aids in memory consolidation. Functional reciprocal connectivity between the ACC and the A1 has also been shown, particularly for auditory attention or by way of ACC-dependent modulation of spontaneous activity in the A1 (Benedict et al., 2002; Hunter et al., 2006; Mulert et al., 2007). The cingulate cortex also has extensive connections with the prefrontal cortex, amygdala, thalamus, and striatum, receives extensive inputs from pain pathways, and contributes to the corticospinal tract (Vogt et al., 1979; Pandya et al., 1981; Finch et al., 1984; Vogt, 2005). The recognized role of the ACC in the pathophysiology of individuals suffering from chronic pain in the absence of nociceptive inputs (Fuchs et al., 2014; Sellmeijer et al., 2018) is consistent with the ACC also playing a significant role in tinnitus, driving the perceived ringing in the absence of auditory inputs (Chen et al., 2018). Enhanced activity in the cingulate cortex has also been observed in tinnitus distress (Vanneste et al., 2010; Vanneste and De Ridder, 2013).

## Central Changes in Traumatic Noise-Exposed Animals

### Amygdala

Supplementary Table S1 compares the species and methodologies used and briefly summarizes the results obtained examining aberrant plasticity in six experimental animal models of traumatic noise exposure in the amygdala at time points ranging from 45 min through 40 days post-exposure.

It was shown in Syrian golden hamsters that traumatic noise exposure with a 10 kHz tone at 125–127 dB SPL (decibels Sound Pressure Level) presented to the left ear for 4 h significantly increased expression of c-fos immunoreactivity in CeA, LA and basolateral amygdala (BLA; Zhang et al., 2003). No laterality of differences were observed other than in CeA, where concentrations were higher ipsilateral to the exposed ear 33–40 days post-exposure. Animals exposed to traumatic noise were also screened for behavioral evidence of tinnitus using a conditioned lick suppression/avoidance paradigm. Hearing loss was measured by examining thresholds of the acoustically evoked brainstem responses (ABR), which reflect differences in sound-induced auditory nerve activity and brainstem activity within the ventral cochlear nucleus (CN) and the ascending auditory nuclei. Zhang et al. (2003) also reported that expression of c-fos in a “stimulated,” non-traumatic noise-exposed group (10 kHz tone, 80± dB SPL for 45 min) was increased in CeA, LA, and BLA immediately after this non-traumatic noise exposure, although the magnitude of this increase in this reduced exposure condition was not quantified nor compared across hemispheres. Neuronal expression of the immediate-early gene (IEG) product, c-fos, is one of a family of genes that are rapidly and transiently activated and translated in response to particular stimuli (Gall et al., 1998) and are widely used as a marker of neuronal activity. IEGs represent an early transcriptional and translational response mechanism activated in the first round of cellular responses to stimuli. C-fos plays a role in neuronal plasticity and is expressed when processing or associating novel sensory stimuli (Tischmeyer and Grimm, 1999). The immunocytochemical findings of Zhang et al. (2003) indicate that multiple amygdalar nuclei respond strongly to sound (traumatic and non-traumatic) at early (non-traumatic noise: c-fos upregulation observed immediately after sound exposure) and later time points (traumatic noise: c-fos upregulation observed at time points spanning over a month).

Wallhäusser-Franke et al. (2003) also investigated the effects of noise exposure on c-fos expression in the amygdala after freely behaving Mongolian gerbils were exposed to acute impulse noise exposure. A toy pistol was fired once close to each ear at a reported intensity level of 136–142 dB SPL. Gerbils were sacrificed at varying times post-impulse noise exposure (1, 3, 5 or 7 h). C-fos expressing cells were present bilaterally in MeA, LA and BLA, with the highest expression observed 1 h after noise exposure. C-fos immunoreactivity was only seen in CeA nuclei 7 h post-noise exposure, indicating a non-uniform increase in neuronal excitability in different amygdalar nuclei. Wallhäusser-Franke et al. (2003) also compared c-fos expression in other limbic brain regions, but reported the greatest IEG immunoreactivity was observed in the amygdala after acute impulse noise exposure.

C-fos immunoreactivity was, again, explored in relation to noise exposure by Mahlke and Wallhäusser-Franke (2004) in freely behaving Mongolian gerbils. Gerbils were acutely exposed to narrowband (1/3 octave) white noise (NBW) of 80 ± 5 dB SPL centered on either 1 or 8 kHz with a rise/fall time of 5 ms followed by an 800 ms pause for 10 min. Gerbils were perfused 3 h post-noise stimulation, and higher levels of c-fos expression were observed in LA, but rarely in CeA and minimally in MeA regardless of the noise condition. In addition to c-fos, arg 3.1 (activity-related cytoskeletal protein; also commonly referred to as “Arc”; henceforth, the term “Arc” will be maintained for consistency) immunoreactive neurons were also quantified. Arc is involved in long-term memory consolidation and synaptic plasticity (Plath et al., 2006). Arc mRNA and protein levels are mobilized by intense synaptic activity in glutamatergic neurons in an NMDA-receptor-dependent manner (Link et al., 1995; Steward and Worley, 2001). Arc binds to actin, is trafficked to dendrites, and accumulates at sites of synaptic activity where it is locally translated and induces homeostatic scaling of AMPA receptors and cellular structural modifications (Shepherd et al., 2006). Similar to their findings for c-fos, Mahlke and Wallhäusser-Franke (2004) found that NBW exposure increased Arc expression in LA 3 h post-exposure, with minimal expression observed at that time point in CeA or in MeA. Overall, they found that NBW did not elicit as much immunoreactivity as treatment with salicylate (results for salicylate treatment reported below). Moreover, they found that gerbils passively exposed to ambient background noise displayed comparable results in c-fos and Arc expression to gerbils exposed to NBW.

Singer et al. (2013) investigated BLA changes in Arc regulation in a rat model of tinnitus after varying intensities of sound exposure [80 dB SPL (a non-damaging condition) or more intense 100, 110 or 120 dB SPLs at 10 kHz]. Rats were bilaterally exposed to these sounds under anesthesia for 1–2 h, then sacrificed 6–30 days post-exposure. ABR waveform correlation factors were calculated, and the hearing thresholds of a subset of the animals were taken 6–14 days post-exposure. A further subset of animals was behaviorally trained and analyzed for tinnitus perception using an operant conditioned foraging task. A trend toward increased Arc mRNA expression was reported in the BLA of animals exposed to 110 dB SPL for 1–2 h. A separate group exposed to 120 dB SPL for 1–2 h exhibited behavioral evidence of tinnitus; on day 14 after this initial noise exposure, these rats expressed reduced Arc mRNA and reduced Arc protein expression in BLA, similar to levels observed in controls. To better characterize this finding, the number of CtBP2/RIBEYE-positive particles in ribbon synapses of the IHC was also measured. Quantification of CtBP2/RIBEYE-positive particles allowed assessment of the degree of deafferentation/degeneration of cochlear hair cells, nerve terminals, and the connecting synapses (Khimich et al., 2005). These findings were related to the integrity of the ABR, and linked to Arc expression in BLA: Singer et al. (2013) observed that a failure to up-regulate Arc occurs after severe ribbon loss and is associated with reduced ABR waves and with behavioral evidence of tinnitus. Singer et al. (2013) also utilized the social stressor paradigm to elevate corticosterone (CORT) levels in rats exposed to 120 dB SPL for 1 h. The social stressor caused CORT levels to elevate within 48 h after the social stressor. Fourteen days after, when hearing capacity was analyzed, the mean ABR wave of the stressed animals showed more consistent maintenance of ABR waveforms compared to controls, indicating that CORT elevation may enable a more stable and persistent responsiveness to sound signals than otherwise would have been achieved. Noise trauma was given 2 days after stress priming Arc expression in groups with either moderate or high CORT levels at time of trauma. Those with significantly lower Arc expression in BLA were animals with higher CORT, while Arc up-regulation was observed with moderate CORT levels. This suggests that moderate stress may positively influence VIIIth nerve IHC ribbon numbers by recruiting Arc up-regulation proportional to the extent of damage at the IHC synapse following acoustic trauma, whereas very high or very low CORT levels at the time of trauma promote less protection from ribbon loss, leading to persistently reduced ABR wave sizes and a failure to up-regulate Arc expression.

Electrophysiological changes in BLA resulting from exposure to a 10 kHz tone at 105 dB SPL for 3 h have also been investigated using multichannel electrode arrays in rats with and without behavioral evidence of tinnitus at 2 and 6 week post-traumatic noise exposure by Zhang et al. (2016). Behavioral evidence of tinnitus was assessed at week 5–6 post-noise exposure using a gap-prepulse inhibition of the acoustic startle paradigm (also referred to as a gap detection acoustic startle reflex). This paradigm, originally suggested for studies involving animal models of tinnitus by Turner et al. (2006), provides a relatively fast screening for evidence of tinnitus in animals by assuming that if a background signal is perceptually similar to the animal’s perception of tinnitus, then the animal will show poorer detection of a silent gap embedded in the background signal. Control animals experience the silent gap, and their reflexive startle responses on trials with this gap are lower in amplitude than on trials without the silent gap; in experimental animals the tinnitus “fills in the silence” on some or all gap trials, and their responses are similar to those of controls on non-gap trials. Zhang and colleagues also utilized a conditioned lick suppression paradigm (Pace et al., 2016) to assess behavioral evidence of tinnitus at 7 week post-noise exposure. Here, water-deprived rats were trained to lick a water spout when they heard narrowband sounds in order to receive water as a reward. If rats licked during silent trials, they received a mild foot shock to train them to only lick during narrowband noise trials. Post-noise exposure, rats were considered to have evidence of tinnitus if they increased licking behaviors during silent trials relative to baseline performance. Zhang et al. (2016) found significantly higher spontaneous BLA firing rates in rats with behavioral evidence of noise-induced tinnitus at 6 week post-noise exposure, but no alteration in firing observed at 2 week post-noise exposure when compared with rats that were exposed to the same noise that did not present with behavioral evidence of tinnitus. Essentially, 2 weeks after rats were exposed to traumatic noise, all had significant increases in spontaneous BLA firing rates, independent from behavioral evidence of tinnitus (assessment of behavioral evidence was only reported for weeks 5–7 post-noise exposure); however, spontaneous firing rates from rats without evidence of tinnitus (as reported for weeks 5–7) returned to basal levels 6 week post-noise exposure, whereas spontaneous BLA firing rates of rats presenting with evidence of tinnitus remained elevated. In the same study, Zhang et al. (2016) also reported significantly higher spontaneous neural synchrony in BLA of rats with behavioral evidence of tinnitus as compared to rats without evidence of tinnitus at both 2 and 6 week post-traumatic noise exposure, indicative of additional BLA plasticity in tinnitus.

Kapolowicz and Thompson (2016) also investigated changes in the amygdala after traumatic noise exposure. After bilaterally exposing freely behaving rats to 1 h of traumatic noise (16 kHz, 115 dB SPL), an upregulation of Arc protein expression was observed in the amygdaloid complex within 45–60 min post-noise exposure. To assure that changes were due to traumatic noise exposure rather than to the novelty of exposure to a sustained sound itself (novelty often up-regulates Arc expression in other brain regions), Kapolowicz and Thompson (2016) also tested the effect of non-traumatic noise exposure (16 kHz, 70 dB SPL) for the same duration and found no significant change in Arc protein expression for this condition relative to controls. In an attempt to understand if the observed upregulation of Arc resulting from traumatic noise exposure was linked to disinhibition, potential changes in GAD expression resulting from traumatic noise exposure were also quantified. GAD is the biosynthetic enzyme that catalyzes the decarboxylation of glutamate into the major inhibitory neurotransmitter, GABA, and it is expressed in two isoforms: GAD 65 and GAD 67 (Erlander et al., 1991). Kapolowicz and Thompson (2016) reported no significant changes in GAD 65 + GAD 67 after exposure to acute high-intensity noise. In order to see if the observed upregulation of Arc protein expression was linked to a stress response, Kapolowicz and Thompson (2016) also tested circulating serum CORT levels in rats exposed to traumatic noise at the same time point as animals were sacrificed (45–60 min post-sound exposure), but found no change in CORT when compared to controls at this early time point post-noise exposure. In the same study, the authors were also interested in whether D-cycloserine (DCS), an NMDA NR1 receptor partial agonist, would be able to reduce or prevent traumatic noise-related plastic changes in Arc protein expression. They found that, when intraperitoneally (i.p.) injected 15 min prior to the start of sound exposure, acute traumatic noise-exposed rats treated with DCS did not exhibit an increase in Arc expression. Although the rats in this study were sacrificed within 1 h post-noise exposure, future research could monitor rats for behavioral evidence of tinnitus across relevant time intervals to determine how effective DCS could be in preventing, delaying, or reducing the manifestation of tinnitus.

### Hippocampus

Supplementary Table S2 compares the species and methodologies used and briefly summarizes the results obtained examining aberrant plasticity in six experimental animal models of traumatic noise exposure in the hippocampus at multiple time points from 30 min to 10 weeks post-exposure.

Goble et al. (2009) reported plasticity-related changes in hippocampal CA1 neurons in freely behaving rats after acute bilateral noise exposure of 4 kHz at 104 dB SPL for 30 min. They reported that previously stable CA1 place cell responses were immediately altered after noise trauma and never re-stabilized to their original firing properties while being monitored and recorded for 24 h post-noise exposure. This noise-induced plasticity in place-field location specificity is noteworthy since prior work has demonstrated extreme stability of location-specific firing in the absence of specific changes in the spatial environment, stability persisting for periods of at least months at a time (Thompson and Best, 1989, 1990). After noise exposure sufficient to induce tinnitus, Goble et al. (2009) observed rapid (i.e., within <1 h) changes in place-field position, in spatial location correlation values, in grand-mean firing rates, in in-field and out-of-field firing rates, and in peak-firing rates compared to controls. These effects persisted for >24 h post-noise exposure and resulted in long-term plasticity in the spatial firing correlates of hippocampal neurons. Results from this study showed that evidence for plasticity resulting from traumatic noise exposure occurs in the hippocampus very rapidly after traumatic noise exposure and leads to long-term changes in hippocampal functional correlates.

Kraus et al. (2010) investigated whether neurogenesis in the hippocampal dentate subgranular zone was affected by a unilateral exposure to an acute 12 kHz tone at 126 dB SPL for 2 h in a rat model of tinnitus. When the animals were sacrificed 10 weeks post-noise exposure, they found that doublecortin (DCX; used for immunolabeling neuronal precursor cells) was reduced. DCX is a microtubule-associated protein expressed in neuronal precursor cells, but not in glial cells nor in neural stem cells from which the precursor cells develop. Upon migration and maturation into functional neurons, DCX expression is downregulated (Brown et al., 2003); DCX is thus a viable marker of neurogenesis. Immunolabeling of Ki67 (to label proliferating cells) was also reduced from exposure to the same acoustic trauma. Ki67 is a mitosis marker for cell proliferation that is expressed during all active phases of the cell cycle but absent in differentiated neurons (Scholzen and Gerdes, 2000). Taken together, Kraus et al.’s (2010) results were indicative that noise trauma impairs neurogenesis in the dentate of the hippocampus. All animals exposed to traumatic noise also exhibited sensory hair cell loss in their exposed ear. These animals were also tested for behavioral evidence of tinnitus using the gap-prepulse inhibition of the acoustic startle paradigm (Turner et al., 2006), and only a subset of rats presented behavioral evidence of tinnitus. While noise trauma specifically impaired later hippocampal neurogenesis, it did not necessarily lead to behavioral evidence of tinnitus in the gap-startle paradigm used.

Given the previous cited results showing early plastic changes in hippocampus due to traumatic noise exposure as well as others implicating the hippocampus in maintenance of tinnitus (Rauschecker et al., 2010; Roberts et al., 2010, 2013), Zheng et al. (2011) wanted to better characterize how hippocampal function might be impacted by acoustic noise exposure. Specifically, they investigated whether rats that had been exposed unilaterally to a 16 kHz tone at 110 dB SPL for 1 h would exhibit spatial memory deficits. Spatial memory deficits are linked to hippocampal functional impairments (e.g., in humans, Nunn et al., 1999; Guderian et al., 2015; in animals, Morris et al., 1982; Aggleton et al., 1986; Pioli et al., 2014). Using both T-maze and Morris water maze tasks, they found that spatial memory in rats with tinnitus was not impaired 2 months after exposure to acoustic trauma. They also utilized a lick suppression paradigm to confirm behavioral evidence of tinnitus in their rats 2 weeks and again at 10 months post-noise exposure, suggesting that some forms of hippocampal plasticity in tinnitus may not be directly linked to spatial memory impairments.

Singer et al. (2013) also examined whether changes in the hippocampus would be observed 14 days after various levels of noise exposure. They found an increase in Arc mRNA expression in CA1 after exposure to 100 and 110 dB SPL with a 10 kHz tone for 1 or 1.5 h. After 120 dB SPL exposure, however, Arc expression was no different from controls. These results were independent of whether rats were exposed to the noise for 1.5 h or only 1 h. This finding was further characterized by measuring the number of CtBP2/RIBEYE-positive particles in ribbon synapses of the IHC in order to determine the degree of deafferentation. The results were related to the integrity of the ABR and Arc expression in CA1 as in BLA: rats exposed to a 10 kHz tone at 120 dB SPL with behavioral evidence of tinnitus had the most severe IHC ribbon loss and most severe loss of ABR wave correlations (an 80% decline from baseline). Singer et al. (2013) also found that adding a social stressor 2 days prior to traumatic noise exposure raised circulating CORT levels in rats, which was linked to a more consistent maintenance of ABR waveforms compared to non-stressed controls. This effect indicates that CORT elevation allows for more stable and persistent responses to sound, a higher number of IHC ribbons, and greater mobilization of Arc mRNA in limbic regions (BLA and CA1 of the hippocampus). It suggests that moderately elevated circulating levels of CORT may serve a protective mechanism in both the cochlea and limbic regions, whereas high or very low levels of circulating CORT prevents this synaptic protection. This protection hypothesis is one that clearly deserves further study.

Kapolowicz and Thompson (2016) further investigated early plasticity in dorsal hippocampal processing of traumatic noise. Freely behaving rats were bilaterally exposed to either a 16 kHz tone at 115 dB SPL, a 16 kHz tone at 70 dB SPL, or to silence, with exposure for each condition lasting 1 h. Rats were sacrificed 45 min-1 h after cessation of these sound conditions, and Arc protein expression was quantified for dorsal hippocampus; this time interval was selected to capture peak expression of transient Arc protein (McIntyre et al., 2005; Czerniawski et al., 2011; Holloway-Erickson et al., 2012). They found that Arc was upregulated after traumatic noise exposure (115 dB) but not after non-traumatic (70 dB) noise exposure. The authors also investigated potential changes in inhibition, using the biomarker GAD, after traumatic noise exposure. They found no significant changes in GAD 65 + 67 protein expression in the dorsal hippocampus. Kapolowicz and Thompson (2016) also examined whether the partial NMDA-receptor agonist, DCS, could maintain Arc protein expression at basal levels in the dorsal hippocampus after exposure to acoustic trauma: DCS did not prevent changes in Arc mobilization. The 6 mg/kg (ip) dose of DCS administered by Kapolowicz and Thompson (2016) was previously shown to increase hippocampal intrinsic excitability and Arc protein expression in non-noise exposed rats (Donzis and Thompson, 2014) as well as facilitate hippocampally-dependent memory in other species (Thompson et al., 1992; Thompson and Disterhoft, 1997). Although DCS functions as a partial agonist, it was predicted that, when paired with traumatic noise exposure, DCS would instead function antagonistically due to NMDA binding sites being saturated with less potent DCS competing off serine, the full agonist for the NMDA receptor, as observed in amygdala post-traumatic noise exposure with the same dosage. Relative to controls, Kapolowicz and Thompson (2016) observed elevated levels of Arc expression in dorsal hippocampus after treatment with DCS paired with traumatic noise exposure, similar to when rats were exposed to traumatic noise alone (without DCS treatment). Given that DCS was able to prevent elevation of Arc in the amygdala of traumatic noise-exposed rats treated with DCS, unlike for the dorsal hippocampus, future research should compare these results of DCS treatment with manipulations using serine, the endogenous ligand for NR1 subunits of NMDA receptors. Such an investigation would further address whether NMDA-receptor mediated plasticity is required at this early stage in tinnitus-related plasticity in the amygdala and in hippocampus.

Previous *in vitro* recordings found that long-term potentiation is inhibited within the CA1 region of the hippocampus after *in vivo* long-term exposure to high-intensity noise (Cunha et al., 2015). To further characterize this result, work from the same lab found that post-burst hyperpolarizations are increased due to a decrease in the h (hyperpolarization-activated) current; they also observed an increase in firing of CA1 pyramidal neurons, but the mechanism for this increase in excitability remained unknown (Cunha et al., 2018). Recently, Cunha et al. (2019) continued to better characterize this finding that high-intensity sound effects long term potentiation in CA1 hippocampal neurons using the same paradigm as their previous work, with rats exposed to high-intensity broadband noise (110 dB, 2–15 kHz, with a peak at 7 kHz) twice daily for 10 days, for 1 min each session. This pattern of noise exposure was meant to emulate exposure to a loud occupational/recreational sound in an animal model, as occupational and recreational sounds are common causes of auditory-related issues reported from both younger (Lercher et al., 2003) and older (Helfer et al., 2011) populations. Using *in vitro* whole-cell patch-clamp recordings to study synaptic transmission, they found that inhibitory GABAergic transmission is increased within CA1 after high-intensity noise exposure over the course of several days. They found no changes in excitatory glutamatergic activation of AMPA/kainate or NMDA receptors. While GABAergic enhancement is consistent with the observed inhibition of long-term potentiation in this paradigm, Cunha et al. (2019) speculated that this increase in inhibition could be compensatory and protective against loud sound exposure.

### Cingulate

Supplementary Table S3 compares the species and methodologies used in two animal models exposed to traumatic noise exposure and briefly summarizes the results obtained examining potential changes in the cingulate cortex at different time points from 1 to 7 h post-exposure. The cingulate cortex is a limbic structure that has been less well-characterized regarding its prospective involvement in potentially tinnitus-inducing maladaptive plasticity. In much of the cognitive neuroscience literature (e.g., Stevens et al., 2011), important distinctions are drawn between anterior and posterior cingulate cortex, however in animal models exposed to traumatic noise, such a distinction is not always stipulated, or the focus is adhered to strictly the ACC. Henceforth in the present review, distinctions will be overtly stated if the distinction was provided in the original research. Otherwise, the general term of “cingulate cortex” will be used.

Wallhäusser-Franke et al. (2003) investigated how c-fos expression could be altered in various classic and non-classic auditory structures, including the ACC. They found that c-fos immunoreactivity in cingulate cortex was elevated in gerbils 1 h after being bilaterally exposed to impulse noise (136–142 dB SPL) but was reduced to control levels within 7 h post-traumatic noise exposure. Rapid and transient c-fos induction is associated with exposure to novel sensory stimuli (Tischmeyer and Grimm, 1999). The C-fos expression has been used to identify activation in various auditory regions, with upregulation found in relation to the significance of the acoustic signal (e.g., Carretta et al., 1999). Aligning with previously reported results, Wallhäusser-Franke et al.’s (2003) results described here are indicative that c-fos is also responsive to auditory stimuli within the ACC shortly after exposure to a traumatic sound.

Mahlke and Wallhäusser-Franke (2004) investigated potential changes in Arc and in c-fos immunoreactivity within the ACC of gerbils 3 h after 10 min of exposure to approximately 80 dB SPL NBW centered at two different frequencies (8 kHz or 1 kHz). They detected an upregulation of Arc within the ACC for both frequencies of noise exposure, but no significant differences due to treatment condition. Although they did not statistically analyze the expression of c-fos across groups, they also observed increases in c-fos immunoreactivity for all treatment conditions, with c-fos expression always outnumbering Arc expression, though the magnitude of this difference was not given.

## Central Changes in Animals Treated With Salicylate

### Amygdala

Supplementary Table S4 lists the species and methodologies utilized in five animal models exposed to sodium salicylate and briefly reports on the results obtained from these studies regarding potential changes in the amygdala at time points from 1 to 5 h post-exposure.

In the thorough study by Wallhäusser-Franke et al. (2003), the effect of impulse noise exposure on c-fos immunoreactivity was directly compared to the effects of treatment with either a high (350 mg/kg) or a low (50 mg/kg) dose of sodium salicylate in gerbils 3 h post-injection. The overall expression of c-fos in various brain regions revealed that c-fos immunoreactivity was lower after impulse noise exposure than after a high dose injection of salicylate. Within different amygdaloid nuclei (CeA, LA, BLA, and MeA), an abundance of labeled cells were observed bilaterally after a high dose of salicylate injection, whereas treatment with a low dose did not alter labeling from controls. The highest densities of c-fos expression were observed in the CeA.

Mahlke and Wallhäusser-Franke (2004) compared both Arc and c-fos immunoreactive expression resulting from either NBW exposure or salicylate treatment (350 mg/kg) in gerbils. Salicylate treated gerbils were further subdivided into groups also exposed to either ambient background noise or silence. Within the amygdaloid complex, Arc and c-fos immunoreactive neurons were substantially increased 5 h after salicylate injections compared to NBW stimulation, especially in CeA (where Arc and c-fos immunoreactive neurons were found exclusively after tinnitus-inducing treatments): Salicylate treatment combined with exposure to ambient background noise and salicylate treatment paired with exposure to silence led to strong Arc expression in CeA (mostly the lateral subdivision) as well as in LA, but expression was negligible in MeA. In comparison, gerbils exposed to NBW exhibited Arc staining of neurons in LA, but negligible staining in both CeA and MeA nuclei. Saline treatment paired with ambient background noise and saline treatment paired with silence showed only negligible expression of Arc in LA and none in CeA and MeA nuclei. After salicylate (both with and without ambient background noise), the amygdaloid complex presented with many more c-fos expressing neurons in CeA and LA nuclei than was observed in gerbils exposed to NBW. In comparison, after NBW exposure (paired with either ambient background noise or silence), c-fos immunoreactive neurons were always present in LA (a comparable expression to that seen for Arc) but rarely in CeA. Overall, higher levels of c-fos expression were observed compared to Arc expression, but the c-fos expression in MeA was minimal in all treatment groups.

Chen et al. (2012) investigated the effects of treatment with salicylate (300 mg/kg) on LFP and frequency receptive fields of neurons in LA in rats immediate after, 1 h, and 2 h post-treatment. They found that salicylate increased the amplitude of the LFP, making it hyperactive to sounds greater than 60 dB SPL. They also found that the frequency receptive fields of multiunit clusters in LA were also dramatically altered by salicylate: Neuronal activity at frequencies below 10 kHz and above 20 kHz was depressed at low intensities, but greatly enhanced for stimuli between 10 and 20 kHz (frequencies near the observed pitch of salicylate-induced tinnitus in rats). These frequency-dependent changes caused the frequency receptive fields of many LA neurons to migrate towards responses to 10–20 kHz stimuli (i.e., tonotopic reorganization), thereby amplifying activity in this frequency band. They also observed that the infusion of salicylate (20 ml, 2.8 mM) directly into LA enhanced sound-evoked activity in AC, i.e., increased LFP amplitude and enhanced AC neuronal activity at these same mid-frequencies, associated with the pitch of salicylate-induced tinnitus in rats.

In a separate study, Chen et al. (2014) further investigated changes in the excitability of LA neurons in rats 2 h post-treatment with salicylate (200 or 250 mg/kg). To identify electrophysiological changes within LA, sound-evoked LFPs and multiunit discharges were recorded before and after salicylate treatment. A subset of rats was trained on a two-alternative forced-choice identification task to test for behavioral evidence of tinnitus resulting from the salicylate treatment. Rats treated with doses of 200 and 250 mg/kg of sodium salicylate showed suprathreshold neuronal hyperexcitability in LA. Salicylate treatment also shortened the temporal response in LA. This salicylate-induced hyperactivity in LA indicates plastic changes occurring within LA during induction and early stages of tinnitus. Physiologically, salicylate treatment significantly enhanced sound-evoked neural activity in LA. The authors noted that the enhancement of sound-evoked activity occurred predominantly at the mid-frequencies [consistent with their findings in Chen et al. (2012), and again reflecting shifts of receptive fields of LA neurons towards the mid-frequency range post-treatment]. The increased number of mid-frequency neurons led to a relatively higher number of total spontaneous discharges in the mid-frequency range, regardless of whether or not the mean discharge rate of each LA neuron increased. Chen et al. (2014) speculated that this tonotopical overactivity in the mid-frequency range in quiet can potentially lead to a tonal sensation within this same range (i.e., tinnitus). The authors also suggested that this plasticity in LA may also contribute to the negative effect that many patients associate with their tinnitus.

Chen et al. (2015) additionally hypothesized that enhanced functional connectivity between hippocampal and auditory brain regions provides a substrate for assigning a spatial location to a phantom sound (findings summarized below), while coordinated activity between specific auditory areas and the amygdala may draw attention to and add emotional salience to neural activity in the auditory pathway. Thus, functionally coordinated activity within hippocampal, amygdalar and cortical networks may be essential for bringing tinnitus into consciousness. Although it is beyond the scope of this present review to describe results observed in classic auditory structures, Chen et al. (2015) treated rats with sodium salicylate (300 mg/kg) and tested responses 2 h post-injection to investigate their hypothesis (A separate group of rats was treated with the same dose of salicylate and later tested for behavioral evidence of tinnitus using a two-alternative forced-choice paradigm to assure that their drug treatment could effectively serve as an animal model of tinnitus). Chen et al. (2015) found that salicylate vigorously amplified sound-evoked neural responses in LA *via* changes in LFP amplitude-intensity functions. These electrophysiological changes were compared with resting-state fMRI patterns, which revealed hyperactivity in the auditory network (i.e., inferior colliculus (IC), medial geniculate, and A1) with connections to amygdala and other regions *via* amplitude of low-frequency fluctuations (ALFF). Functional connectivity revealed enhanced coupling within the auditory network and other regions including the amygdala, further strengthening their hypothesis.

### Hippocampus

Supplementary Table S5 summarizes the species and methodologies utilized in five animal models exposed to sodium salicylate and briefly reports on the results obtained from these studies regarding potential changes in the hippocampus at multiple time points from 2 h to up to 39 days post-exposure.

Wallhäusser-Franke et al. (2003) investigated the effects of treatment with either a single high dose (350 mg/kg) or a single low dose (50 mg/kg) injection of sodium salicylate in Mongolian gerbils 3 h post-treatment. They found bilateral expression of c-fos in both dentate and subiculum of the hippocampus in gerbils injected with the high dose of salicylate (a dose shown above to cause behavioral evidence of tinnitus in several animal models). In all regions assessed by Wallhäusser-Franke et al. (2003), including hippocampal regions, it was found that the greatest immunoreactivity was observed after this high dose treatment with salicylate when compared to the lower dose treatment group or to the group exposed to loud impulse noise.

Gong et al. (2008) examined potential changes in extrinsic excitation and inhibition in cultured rat neurons from the CA1 hippocampus during the *in vitro* bath application of sodium salicylate. Extracellular recordings showed that sodium salicylate enhanced the amplitude of evoked population spikes in a dose-dependent manner. Salicylate at 1 mM caused a leftward shift of the evoked EPSP curve, indicating an excitatory potentiation. This effect was reversible after washout. Salicylate had no effect on basal field EPSPs, suggesting that synaptic input remained unchanged during drug treatment. These results indicate that salicylate enhances the likelihood that EPSPs would cross the threshold to generate action potentials, reflecting increased excitation of CA1 neurons. Gong et al. (2008) also investigated possible changes in inhibition, and found that salicylate reduced GABAergic inhibition, leading to increased CA1 neuronal excitation. Both evoked (eIPSCs) and spontaneous (mIPSCs) were suppressed by salicylate with no change in input resistance. Only the amplitude, but not the frequency, of mIPSCs was reduced. Similarly, salicylate directly suppressed GABAaR-mediated whole-cell currents in cultured CA1, consistent with the effects on the amplitudes of mIPSCs and eIPSCs. Gong et al. (2008) concluded that salicylate reduces GABAergic transmission *via* suppression of GABAaR-mediated responses. These acute effects on inhibition were fully reversible by washout.

Chen et al. (2014) investigated the effect of treatment with salicylate (either 200 or 250 mg/kg) on rats 2 h post-treatment. Rats treated with both these doses showed suprathreshold hyperexcitability in the hippocampus (and in the amygdala, as detailed earlier). Again, this salicylate-induced hyperactivity is a consistent form of plastic change in the hippocampus. This electrophysiological plasticity was significant in recordings of sound-evoked LFPs and of multiunit discharges 2 h after treatment. Tinnitus-inducing treatment with salicylate significantly and rapidly enhanced sound-evoked neural activity in hippocampus. The enhancement of sound-evoked activity occurred predominantly at the mid-frequencies (as in the LA, described above), likely reflecting shifts of neuronal responses towards mid-frequency ranges post-salicylate treatment. Chen et al. (2014) explained that the increased number of mid-frequency responsive neurons would lead to a relatively higher number of total spontaneous discharges in the mid-frequency range, even though the mean discharge rate of each individual neuron need not increase. As also observed in LA, this tonotopical plasticity (frequency shift and hyperactivity) within the hippocampus in the mid-frequency range in quiet environments could lead to tonal mid-frequency sensations presenting as tinnitus. Again, Chen et al. (2014) tested a subset of rats given salicylate treatment for behavioral evidence of tinnitus and found that the treatment inducing early hippocampal plasticity also produced behavioral signs of tinnitus in this animal model.

Wu et al. (2015) investigated the effects of acute and chronic treatment with sodium salicylate (300 mg/kg) on CA1 hippocampal mRNA and on protein expression of Arc, Early growth response 1 (Egr-1), and NMDA receptor subunit 2B (NR2B). To reiterate, Arc expression has been shown to be involved mechanistically in long-term memory consolidation and synaptic plasticity (Plath et al., 2006). Egr-1 has been shown to be essential for the persistence of late-phase long-term potentiation within the hippocampus as well as the consolidation of several forms of long-term memory (Davis et al., 2010; Penke et al., 2014; Duclot and Kabbaj, 2017). NR2B is associated with NMDA receptor activation *via* glutamate binding, which is critical for age-dependent thresholds of plasticity and memory formation (Tang et al., 1999). Wu et al. (2015) acutely treated one group of rats with a single dose (300 mg/kg) of salicylate, and these rats were sacrificed 2 h post-treatment. Chronically treated groups were given salicylate once daily for 10 consecutive days, and sacrificed either on day 11 or put into in one of two recovery groups which were sacrificed on either day 25 or day 39. Wu et al. (2015) found that expression of Arc mRNA and protein were up-regulated after either acute or chronic salicylate treatments. Specifically, they reported an upregulation of Arc mRNA and protein expression after acute treatment, which further increased significantly for rats chronically treated with salicylate for 10 days. They also observed an increase in the number of presynaptic vesicles, the thickness of postsynaptic densities, and an increase in synaptic interface curvature (i.e., expansion of synaptic area) in the group sacrificed on day 11. They also observed an upregulation of Egr-1 and NR2B mRNA and protein levels solely for rats chronically treated with salicylate. By day 25, expression for all three biomarkers returned to basal levels (intermediate intervals between 11 and 25 days post-treatment were not assessed).

As previously detailed, Chen et al. (2015) were interested in testing for enhanced functional connectivity between hippocampus and auditory areas in the acute salicylate-treated rat model of tinnitus. Chen and colleagues speculated that enhanced functional connectivity between the hippocampus and auditory areas might provide a substrate for assigning a spatial location to a phantom sound. In their experiments, rats were treated with sodium salicylate (300 mg/kg), and a separate group of rats given this same treatment were tested for behavioral evidence of tinnitus using a two-alternative forced-choice paradigm. Rats were tested before (baseline) and 2 h post-injection with MRI BOLD data acquisition. Similar to their results reported above for the amygdala, Chen et al. (2015) showed enhanced coupling between the auditory network and hippocampus.

### Cingulate

Supplementary Table S6 displays the species and methodologies utilized in three animal models exposed to sodium salicylate and summarizes the results obtained from these studies regarding potential changes in the cingulate at different time points from 2 to 5 h post-exposure.

The cingulate cortex, another key limbic region investigated by Wallhäusser-Franke et al. (2003), showed elevation of c-fos labeled neurons 3 h after a high dose injection of salicylate (350 mg/kg) in gerbils. The authors noted this upregulation of c-fos specifically in the ACC subregion. This region has been implicated in pain processing (Hudson, 2000) and is often co-activated with the amygdala in affectively stressful autonomic responses.

Plasticity in the ACC after high dosage of salicylate was also reported by Mahlke and Wallhäusser-Franke (2004). After a 350 mg/kg dosage of sodium salicylate, they observed an upregulation of both Arc and c-fos expressing neurons in gerbils 5 h post-injection. C-fos expressing neurons outnumbered Arc expressing neurons after this high dose treatment with salicylate (They also reported a similar finding in the ACC after traumatic noise exposure, see above). They proposed that since layers 2 and 3 of the cingulate cortex are directly innervated by primary A1 (Budinger and Scheich, 2009), and they found that Arc immunoreactivity paralleled their findings of increases in Arc expression in A1, then this might entail that A1 activation may exert direct influence on the cingulate cortex.

A third group (Chen et al., 2014) reported no changes in cingulate cortical neuron excitability or frequency response after treatment with either 200 or 250 mg/kg of sodium salicylate, despite behavioral evidence of tinnitus observed in a subset of treated rats trained on a two-alternative forced-choice identification task. These experiments assessed changes in sound-evoked LFPs and in multiunit discharges 2 h post-treatment. Although Chen et al. (2014) observed no evident changes in cingulate cortex in this paradigm, hyperactivity was observed in both LA and hippocampus at this early time point, as described above.

## Concluding Remarks and Future Directions

In a variety of different animal models of tinnitus reviewed here, hyperexcitability, including increases in IEG expression, increased firing activity, and increased sensitivity to inputs, is consistently observed in multiple limbic (i.e., non-classical auditory) regions. The amygdala, the hippocampus, and the cingulate cortex are rapidly responsive to acoustic trauma, beginning immediately after cessation of noise exposure, and continue to exhibit plasticity well beyond the period after noise exposure ends. These same regions are also rapidly responsive to treatment with sodium salicylate sufficient to induce behavioral signs of tinnitus. These results of both early and sustained plasticity within these limbic regions support the hypothesis that non-classical auditory (i.e., limbic) regions play a vital role in both the manifestation and the maintenance of this common auditory disorder. Moreover, aberrant enhanced connectivity between limbic and classical auditory structures may contribute not only to the sensory sensation perceived as tinnitus but also to the emotional response to the percept of tinnitus, extending prior sensory gating hypotheses (Rauschecker et al., 2010) that do not address the potential involvement of limbic regions in the initiation of tinnitus. Further evidence for the potential involvement of limbic regions during the initiation phase of tinnitus is also reviewed by Kraus and Canlon (2012).

What currently remains unclear is the role that reduced inhibition plays early in tinnitus in these limbic regions. Considerable evidence of changes in inhibitory neurotransmission resulting from traumatic noise exposure have been well-characterized in classic auditory structures, and is beyond the scope of this review (e.g., Browne et al., 2012; Zheng et al., 2014; Heeringa and van Dijk, 2016). Also well-characterized are changes in inhibition in classic auditory regions resulting from ototoxic treatments such as sodium salicylate (e.g., Wang et al., 2006, 2016; Liu et al., 2007; Wu et al., 2018). However, considerably less research has focused on early changes in inhibition in limbic regions in animal models of tinnitus. This makes it difficult to assess whether reduced inhibition or other mechanisms may drive maladaptive hyperexcitability in different limbic regions in tinnitus. It is thus difficult to assess speculation that inhibitory gating from limbic regions may suppress the tinnitus percept at later time points in the clinical progression of this auditory disorder. At present, three separate investigations on changes in inhibition within limbic regions report conflicting results: one report found no changes in GAD expression within amygdala or hippocampus soon after acute noise trauma (Kapolowicz and Thompson, 2016), whereas Gong et al. (2008) found a decrease in inhibitory transmission in hippocampus after treatment with salicylate. Conversely, a more recent study reported an increase in GABAergic inhibition within the hippocampus after prolonged exposure to high-intensity sound (Cunha et al., 2019). These disparate results come from studies with widely different methodologies, making it difficult to infer a clear role for inhibitory changes within these limbic regions in tinnitus.

Even if a decrease in inhibition contributes to hyperexcitability in some tinnitus models, rectifying such a change may not prevent the chronic tinnitus percept. Specifically, Zheng et al. (2014) found that after exposure to traumatic noise (16 kHz pure tone at 115 dB for 1 h), early (5 mg/kg s.c., 30 min, and every 24 h for five consecutive days post-noise exposure) or late (3 mg/day for 45 week beginning 17.5 week post-noise exposure) treatment with L-baclofen, a GABA-B receptor agonist, failed to prevent development of behavioral evidence of tinnitus in rats. If limbic regions are crucially involved from induction through maintenance of tinnitus, then the better detailed characterization of regional limbic inhibition at multiple time points is critically needed to better assess sensory gating hypotheses related to tinnitus percept and affect.

Tae et al. (2018) used a surface-based vertex analysis of data collected from magnetic resonance imaging to reveal evidence of atrophy in the basal and lateral nuclei of the right amygdala in tinnitus patients compared to controls. The authors speculated that such atrophy was due to patients’ attempting to self-modulate their tinnitus percept by a kind of sensory gating, as suggested by Jastreboff (1990). Tae et al. (2018) also reported a decrease in left hippocampal volume correlating with an increase in tinnitus handicap inventory scores, with smaller hippocampi associated with greater self-reported functional impairment from tinnitus. Given that none of their tinnitus sufferers reported evidence of psychological disorders, Tae and coworkers hypothesized that decreased amygdala and hippocampal volume was directly related to the pathophysiology of tinnitus or sensory gating rather than to emotional distress. These results are insightful, suggesting that individuals who already have reduced limbic regional volume compared to the normal population may be more prone to experience tinnitus, or that specific pathologies in these regions can cause tinnitus.

A recent study supports the latter hypothesis: in mice, moderate noise exposure (80 dB SPL for 2 h/day) meant to simulate environmental noise caused an increase in oxidative stress and tau phosphorylation in hippocampus after just 1 week of exposure, whereas A1 did not become susceptible to such changes until after 3 weeks of noise exposure (Cheng et al., 2016). These results indicate that the hippocampus is more vulnerable at an earlier time point to potentially damaging sounds than classical auditory structures such as the A1. Although Cheng and colleagues did not investigate how increased vulnerability to potentially traumatic sounds may relate to initiation of tinnitus, their results indicate early vulnerability in these limbic regions and warrant further study.

The role played by stress hormones such as cortisol in humans (or corticosterone/CORT in rats) is unclear in limbic neuronal plasticity at early or late time points after tinnitus-inducing acoustic or salicylate treatments. Wallhäusser-Franke et al. (2003) proposed that tinnitus may be an indirect consequence of a loss of auditory input associated with stress. Surprisingly, Kapolowicz and Thompson (2016) found no change in circulating CORT levels in rats 1 h after traumatic noise exposure compared to controls. Singer et al. (2013) in fact reported that heightened stress can result in protection from acoustic trauma. These sparse and disparate experimental results suggest that the role of stress in noise trauma-related plasticity may be complex, and require more thorough investigation. Given data linking altered GABAergic activity with hypothalamic-pituitary-adrenal (HPA) axis function (Bowers et al., 1998; Dent et al., 2007; Cullinan et al., 2008) and that the amygdala and hippocampus are principle brain regions regulating HPA activity following psychological or emotional distress (Herman and Cullinan, 1997; Herman et al., 2003), one focus of future animal models of tinnitus should be to investigate direct relationships between altered inhibitory neurotransmission and hormonal and noradrenergic stress responses, and their impacts on neuronal function in these limbic regions.

As this current review shows, plasticity in both the amygdaloid complex and in the hippocampus, and to a lesser degree in the cingulate cortex, has been characterized in both the initiation and maintenance of experimental models of tinnitus. Prominent descriptions of tinnitus, such as the sensory gating hypothesis, should be updated to reflect evidence that limbic region function is altered not only in tinnitus maintenance but also at much earlier stages. Additional study of cingulate cortex plasticity in tinnitus models is needed to identify additional specific contributions to the development of tinnitus symptomology. The experimental results reviewed here suggest that limbic and other non-classical auditory brain regions are promising targets for researchers seeking a more comprehensive mechanistic understanding of the auditory disorder of tinnitus and may yield useful translational targets for improving treatment.

## Author Contributions

The manuscript was written, edited and expanded by MK and LT. Both authors contributed intellectually and practically to the work.

## Conflict of Interest

The authors declare that the research was conducted in the absence of any commercial or financial relationships that could be construed as a potential conflict of interest.

## Abbreviations

ABR: acoustically evoked brainstem responses
AC: auditory cortex
ACC: anterior cingulate cortex
ALFF: amplitude of low-frequency fluctuations
Arc (arg3.1): activity-related cytoskeletal protein
BA: basal amygdala
BLA: basolateral amygdala
CA: cornu ammonis
CeA: central amygdala
CORT: corticosterone
dB SPL: decibels, sound pressure level
DCS: D-cycloserine
DCX: doublecortin
DG: dentate gyrus
Egr-1: early growth response 1
HPA: hypothalamic-pituitary-adrenal
IEG: immediate-early gene
IHC: inner hair cells
i.p.: intraperitoneally
LA: lateral amygdala
LFP: local field potential
MeA: medial amygdala
MGN: medial geniculate nucleus
NBW: narrow band white noise
NR2B: NMDA receptor subunit 2B.
