# Supplementary material for: Plasticity in Limbic Regions at Early Time Points in Experimental Models of Tinnitus
Source: Front Syst Neurosci. 2020 Jan 24;13:88. doi: 10.3389/fnsys.2019.00088 (PMC6992603; doi:10.3389/fnsys.2019.00088)
Supplement: Supplementary file 1 [file Table_1.pdf]

Authors	Species	Exposure	Induction Time Span	Time Point for Results	Results	Behavioral Testing for Tinnitus
Zhang et al., 2003	Hamster	10 kHz tone at 125-127 dB SPL & 10 kHz tone at 80± dB SPL	4 h (traumatic);<br>45 min (non-traumatic)	Traumatic: several points tested spanning over 1 mth;<br>Non-traumatic: tested immediately post-exposure	Upregulation of c-fos in CeA, LA & BLA in both sound conditions	Conditioned lick suppression/avoidance paradigm (traumatic)
Wallhäusser-Franke et al., 2003	Gerbil	Acute impulse noise exposure at 136-142 dB SPL	Toy pistol fired once close to each ear	1, 3, 5 or 7 h post-noise exposure	C-fos observed in MeA, CeA, LA and BLA, with highest expression 1 h post-noise exposure & only in CeA 7 h post-exposure	n/a
Mahlke & Wallhäusser-Franke, 2004	Gerbil	Narrow band (1/3 octave) white noise of 80 ±5 dB SPL centered at 8 or 1 kHz	10 min	3 h post-noise exposure	Higher levels of c-fos & Arc observed in LA for both frequencies	n/a
Singer et al., 2013	Rat	10 kHz at 80, 100, 110 & 120 dB SPL	1-2 h	6-30 d post-sound exposure	Lower levels of Arc in BLA with evidence of tinnitus with 110 dB SPL exposure for 1 or 1.5 h 14 d post-exposure & elevated Arc in BLA when no tinnitus present;<br>Decreases in Arc observed with ribbon loss, reduced ABR waves and tinnitus;<br>Moderate CORT elevation increases Arc and positively influences ABR wave sizes and IHC ribbon numbers, but high or low CORT levels fail to mobilize Arc, causes reduced ABR waves and IHC ribbon loss	Operant conditioning paradigm tested on a subset of rats
Zhang et al., 2016	Rat	10 kHz tone at 105 dB SPL	3 h	2 & 6 wk post-noise exposure	Higher spontaneous firing rates 6 wk post exposure only with evidence of tinnitus;<br>Higher spontaneous firing rates 2 wk post exposure independent of evidence of tinnitus;<br>At both time points, higher spontaneous neurosynchrony in BLA only with evidence of tinnitus	Gap-prepulse inhibition of the acoustic startle paradigm<br>&<br>Conditioned lick suppression/avoidance paradigm
Kapolowicz & Thompson, 2016	Rat	16 kHz at 115 dB SPL & 16 kHz at 70 dB SPL	1 h	45 min-1 h post-noise exposure	Upregulation of Arc only after traumatic noise exposure (individual nuclei not quantified);<br>Observed changes did not occur with DCS treatment paired with traumatic noise, but DCS alone also increased Arc;<br>No change in GAD 65+67 expression in any condition;<br>No change in CORT levels in either acoustic condition	n/a

Table 1. Effects of noise exposure on amygdala.
