# Supplementary material for: Plasticity in Limbic Regions at Early Time Points in Experimental Models of Tinnitus
Source: Front Syst Neurosci. 2020 Jan 24;13:88. doi: 10.3389/fnsys.2019.00088 (PMC6992603; doi:10.3389/fnsys.2019.00088)
Supplement: Supplementary file 2 [file Table_2.pdf]

Authors	Species	Induction Method	Induction Time Span	Time Point for Results	Results	Behavioral Testing for Tinnitus
Goble et al., 2009	Rat	4 kHz tone at 104 dB SPL	30 min	Immediately and several points up to 24 hr post-noise exposure	Stable CA1 single-unit place cell responses immediately altered post-noise trauma & failed to restabilize to original firing properties for up to 24 h post-noise exposure	n/a
Kraus et al., 2010	Rat	12 kHz at 126 dB SPL	2 h	10 wk post-noise exposure	DCX reduced in subgranular dentate; Ki67 reduced in subgranular dentate	Gap-prepulse inhibition of the acoustic startle paradigm tested on a subset of rats
Zheng et al., 2011	Rat	16 kHz tone at 110 dB SPL	1 h	2 mth post-noise exposure	Spatial memory on T maze and Morris water maze unimpaired after acoustic trauma	Lick suppression paradigm tested at 2 wk and at 10 mth post-noise exposure
Singer et al., 2013	Rat	10 kHz at 80, 100, 110 & 120 dB SPL	1-2 h	6-30 d post-sound exposure	Higher levels of Arc in CA1 after 100 & 110 db SPL with a 10 kHz tone for 1 or 1.5 h; At 120 dB SPL for 1 or 1.5 h, Arc levels were no different from controls if rats had evidence of tinnitus; Arc immobilization observed with increased ribbon loss, reduced ABR waves and tinnitus; Moderate CORT elevation associated with Arc mobilization, more stable ABR waves and lower IHC ribbon loss	Operant conditioning paradigm tested on a subset of rats
Kapolowicz & Thompson, 2016	Rat	16 kHz at 115 dB SPL & 16 kHz at 70 dB SPL	1 h	45 min-1 h post-noise exposure	Upregulation of Arc in dorsal hippocampus only after traumatic noise exposure or DCS paired with traumatic noise; No change in GAD 65+67 expression in any condition; No change in CORT levels in either acoustic condition	n/a
Cunha et al., 2019	Rat	110 dB SPL, broadband noise spanning 2-15 kHz	2 min/day for 10 days	10-14 days post-day 10 of noise exposure	Increase in inhibitory GABAergic transmission from high-intensity noise exposure; No change in excitatory glutamatergic transmission from high-intensity noise exposure	n/a

Table 2. Effects of noise exposure on hippocampus.

Table 2. Effects of noise exposure on hippocampus.
