# Supplementary material for: Plasticity in Limbic Regions at Early Time Points in Experimental Models of Tinnitus
Source: Front Syst Neurosci. 2020 Jan 24;13:88. doi: 10.3389/fnsys.2019.00088 (PMC6992603; doi:10.3389/fnsys.2019.00088)
Supplement: Supplementary file 3 [file Table_3.pdf]

Authors	Species	Exposure	Induction Time Span	Time Point for Results	Results	Behavioral Testing for Tinnitus
Wallhäusser-Franke et al., 2003	Gerbil	Acute impulse noise exposure at 136-142 dB SPL	Toy pistol fired once close to each ear	1, 3, 5 or 7 h post-noise exposure	Increase in c-fos observed 1 h post-noise exposure & reduced to basal levels 7 h post-exposure	n/a
Mahlke & Wallhäusser-Franke, 2004	Gerbil	Narrow band (1/3 octave) white noise of 80 ±5 dB SPL centered at 8 or 1 kHz	10 min	3 h post-noise exposure	Higher levels of c-fos & Arc observed in anterior cingulate cortex for both frequencies;<br>C-fos expression always outnumbered Arc expression for both frequencies	n/a

Table 3. Effects of noise exposure on cingulate cortex.
