# Supplementary material for: Plasticity in Limbic Regions at Early Time Points in Experimental Models of Tinnitus
Source: Front Syst Neurosci. 2020 Jan 24;13:88. doi: 10.3389/fnsys.2019.00088 (PMC6992603; doi:10.3389/fnsys.2019.00088)
Supplement: Supplementary file 4 [file Table_4.pdf]

| Authors | Species | Induction Method (Sodium Salicylate Dosage) | Time Point for Results | Results | Behavioral Testing for Tinnitus |
|---|---|---|---|---|---|
| Wallhäusser-Franke et al., 2003 | Gerbil | 350 mg/kg; i.p. (high dose) or 50 mg/kg; i.p. (low dose) | 3 h post-injection | C-fos expression in CeA, LA, BLA, & MeA after high dose injection;<br>High dose injection increased c-fos expression more so than low-dose injection or loud impulse noise exposure | n/a |
| Mahlke & Wallhäusser-Franke, 2004 | Gerbil | 350 mg/kg; i.p | 5 h post-injection | Arc and c-fos expression were upregulated in CeA and in LA | n/a |
| Chen et al., 2012 | Rat | 300 mg/kg; i.p. or 20 Ml, 2.8 mM infusion | 0, 1 & 2 h post-treatment (systemic) | Increased LFP amplitude in LA (hyperactive to sounds greater than 60 dB SPL);<br>Tonotopic reorganization in LA: multi-unit frequency receptive fields altered (activity at frequencies below 10 kHz or above 20 kHz depressed at low intensities & enhanced between 10 and 20 kHz – frequencies near the pitch of salicylate-induced tinnitus in rats);<br>Infusion into LA enhanced sound-evoked activity in AC (increased LFP amplitude and enhanced AC neuronal activity at mid-frequencies associated near the pitch of salicylate-induced tinnitus in rats | n/a |
| Chen et al., 2014 | Rat | 200 or 250 mg/kg; i.p. | 2 h post-treatment | Tonotopical hyperactivity in midfrequency range in LA;<br>Shortened temporal response in LA | Two-alternative forced choice identification paradigm tested on subset of rats |
| Chen et al., 2015 | Rat | 300 mg/kg; i.p. | 2 h post-injection | Increased LFP amplitude-intensities in LA;<br>Enhanced coupling with auditory network and amygdala | Two-alternative forced choice identification paradigm tested on subset of rats |

**Table 4**. Effects of sodium salicylate on amygdala.
