# Supplementary material for: Plasticity in Limbic Regions at Early Time Points in Experimental Models of Tinnitus
Source: Front Syst Neurosci. 2020 Jan 24;13:88. doi: 10.3389/fnsys.2019.00088 (PMC6992603; doi:10.3389/fnsys.2019.00088)
Supplement: Supplementary file 5 [file Table_5.pdf]

Authors	Species	Induction Method<br>(Sodium Salicylate Dosage)	Time Point for Results	Results	Behavioral<br>Testing for Tinnitus
Wallhäusser-Franke et al., 2003	Gerbil	350 mg/kg; i.p. (high dose) or 50 mg/kg; i.p. (low dose)	3 h post-injection	C-fos expression in dentate and subiculum after high dose injection;<br>High dose injection increased c-fos expression more so than low-dose injection or loud impulse noise exposure	n/a
Gong et al., 2008	Rat	*In vitro* bath application at various doses	Cultured neurons used 12-14 days *in vitro*	Dosage at 1 mM enhanced amplitude of population spikes, caused EPSP potentiation of CA1 neurons;<br>No effect on basal field EPSPs of CA1 neurons;<br>Treatment reduced GABAergic inhibition (both eIPSCs and mIPSCs inhibited by salicylate with no change in input resistence);<br>Only amplitude (not frequency) of mIPSPs reduced;<br>Salicylate directly inhibits GABAaR-mediated whole-cell currents in cultured CA1 similarly to the drug affecting the amplitudes of mIPSCs and eIPSCs;<br>Results indicate salicylate reduces GABAergic transmission via suppression of GABAaR-mediated responses	n/a
Chen et al., 2014	Rat	200 or 250 mg/kg; i.p.	2 h post-treatment	Tonotopical hyperactivity in midfrequency range	Two-alternative forced choice identification paradigm tested on subset of rats
Wu et al., 2014	Rat	300 mg/kg; i.p.;<br>Single dose or chronically treated (once per day for 10 consecutive days)	2 h post-injection (acute);<br>11 days post-injection (chronic);<br>25 days or 39 days post-injection (recovery)	Increase in number of synaptic vesicles, thicker post-synaptic densities, and increase in synaptic interface curvature (chronic treatment);<br>Upregulation of Arc (acute and more so for chronically treated);<br>Upregulation of Egr-1 and NR2B only in chronically treated;<br>Recovery to basal levels on day 25	n/a
Chen et al., 2015	Rat	300 mg/kg; i.p.	2 h post-injection	Enhanced coupling with auditory network and hippocampus	Two-alternative forced choice identification paradigm tested on subset of rats

Table 5. Effects of sodium salicylate on hippocampus.
