# Supplementary material for: Plasticity in Limbic Regions at Early Time Points in Experimental Models of Tinnitus
Source: Front Syst Neurosci. 2020 Jan 24;13:88. doi: 10.3389/fnsys.2019.00088 (PMC6992603; doi:10.3389/fnsys.2019.00088)
Supplement: Supplementary file 6 [file Table_6.pdf]

Authors	Species	Induction Method (Sodium Salicylate Dosage)	Time Point for Results	Results	Behavioral Testing for Tinnitus
Wallhäusser-Franke et al., 2003	Gerbil	350 mg/kg; i.p. (high dose) or 50 mg/kg; i.p. (low dose)	3 h post-injection	Increase in c-fos in ACC; high dose injection increased c-fos expression more so than low-dose injection or loud impulse noise exposure	n/a
Mahlke & Wallhäusser-Franke, 2004	Gerbil	350 mg/kg; i.p.	5 h post-injection	Increase in c-fos and Arc in ACC 5 h post-injection; C-fos expression always higher than Arc expression	n/a
Chen et al., 2014	Rat	200 or 250 mg/kg; i.p.	2 h post-noise exposure	No changes in sound-evoked LFPs or multi-unit discharge rates despite evidence of tinnitus	Two-alternative forced choice identification paradigm tested on subset of rats

Table 6. Effects of sodium salicylate on cingulate cortex.
